# Beauty through Hygiene: Common Sense Ways to Health for Girls

**Published:** 1905-06

**Authors:** 


BEAUTY THROUGH HYGIENE: COMMON SENSE WAYS TO HEALTH FOR GIRLS. By EMMA E. WALKER, M.D. Duodecimo, pp. 301. Illustrated. New York: A. S. Barnes & Company. 1904. (Price, $1.00.)

If one wants a book which will interest a young girl in personal hygiene, a better volume than this cannot be found. It is written in a simple fashion, being not at all technical, the advice it gives is excellent, it embodies very good and modern ideas in regard to fresh air, exercise, deep breathing, etc., and its title is one which will appeal to almost every girl. It can be highly recommended. M. J. F.

